# Perioperative Minimal Induction Therapy: A Further Step toward More Effective Immunosuppression in Transplantation

**DOI:** 10.1155/2012/426042

**Published:** 2012-05-20

**Authors:** Alessia Gennarini, Paolo Cravedi, Maddalena Marasà, Annalisa Perna, Giovanni Rota, Mario Bontempelli, Silvio Sandrini, Giuseppe Remuzzi, Piero Ruggenenti

**Affiliations:** ^1^Mario Negri Institute for Pharmacological Research, Centro Anna Maria Astori, Science and Technology Park Kilometro Rosso, Via Stezzano, 87, 24126 Bergamo, Italy; ^2^Unit of Nephrology, Azienda Ospedaliera Ospedali Riuniti di Bergamo, Bergamo, Italy; ^3^Unit of Pediatric Surgery, Azienda Ospedaliera Ospedali Riuniti di Bergamo, Bergamo, Italy; ^4^Unit of Immunohematology, Azienda Ospedaliera Ospedali Riuniti di Bergamo, Bergamo, Italy; ^5^Unit of Nephrology, Azienda Ospedaliera Ospedali Civili di Brescia, Brescia, Italy

## Abstract

Dual induction with low doses of rabbit anti-human thymoglobulin (RATG) and basiliximab effectively and safely prevented allograft rejection in high-risk renal transplant recipients. To assess whether treatment timing affects efficacy and tolerability, in this single-center, matched-cohort study, we compared posttransplant outcomes in 25 patients and 50 gender-, age-, and treatment-matched reference patients induced with the same course of 7 daily RATG infusions (0.5 mg/kg/day) started before or after engraftment, respectively. All subjects received basiliximab (20 mg) before and 4 days after transplantation, withdrew steroids within 6 days after surgery, and were maintained on steroid-free immunosuppression with cyclosporine and mycophenolate mofetil or azathioprine. Over 12 months after transplant, 1 patient (4%) and 13 reference patients (26%) had acute rejection episodes. One patient and 5 reference-patients required dialysis therapy because of delayed graft function. In all patients circulating CD4+ and CD8+ T lymphocytes were fully depleted before engraftment. Both treatments were well tolerated. In kidney transplantation, perioperative RATG infusion enhances the protective effect of low-dose RATG and basiliximab induction against graft rejection and delayed function, possibly because of more effective inhibition of early interactions between circulating T cells and graft antigens.

## 1. Introduction

Induction therapy with early administration of antilymphocyte antibodies has been introduced into clinical transplantation to modify the host immune response at the time of donor antigen presentation. This approach is aimed at achieving immune hyporesponsiveness of the host expected to translate into a reduced risk of rejection in the posttransplant period [1, 2]. Different antibodies have been used for induction therapy, including monoclonal nondepleting antibodies such as basiliximab and daclizumab, and monoclonal or polyclonal depleting antibodies, such as alemtuzumab or thymoglobulin, respectively [1, 2].

Nondepleting are safer than depleting antibodies, but are less effective [3]. Depleting antibodies may cause severe adverse events including infusion-related cytokine-release syndrome and enhanced risk of CMV reactivations and lymphoproliferative disorders in the long term [4, 5]. To minimize side effects, we introduced in our clinical practice an induction strategy based on rabbit anti-human thymoglobulins (RATGs) administered at very low doses (approximately half the currently recommended doses for induction and one-third to one-fourth of doses administered in the large majority of previous reports [3, 6, 7]). To avoid the risk of insufficient antirejection activity, we integrated this regimen with basiliximab, an anti-interleukin-2 receptor (IL-2R) monoclonal antibody administered with the rationale of inhibiting those lymphocytes eventually surviving low-dose RATG exposure.

In a randomized, prospective study, we showed that this dual induction protocol, compared to the standard single-drug, full-dose RATG regimen, provided the same protective effect against acute allograft rejection in hyperimmune renal transplant patients and in those with delayed graft function (DGF), but was better tolerated [8]. On the basis of these encouraging findings, since October 2004 we applied the dual induction regimen to all new incident patients receiving a kidney transplant at our Institution. Steroids were tapered and withdrawn within 6 days after transplant and patients were maintained on low doses of Cyclosporine A (CsA) and randomly allocated to mycophenolate mofetil (MMF) or azathioprine (AZA), in the setting of an immunosuppressive regimen aimed at achieving effective prevention of allograft rejection with minimized risk of side effects.

In the meantime, however, evidence has become available that, in addition to the adopted treatments, the timing of treatment administration may affect the effectiveness of induction therapy. A randomized clinical trial [9] found that in adult deceased donor renal transplant recipients, RATG infusion started before graft reperfusion was associated with a reduced DGF incidence compared to posttransplant administration of the same RATG regimen [9]. The authors suggested that DGF prevention could be explained by inhibited production and activity of adhesion molecules, which in turn limited leukocyte rolling and adhesion along capillary endothelial surfaces, one of the early interactions between the host and the graft leading to ischemia-reperfusion injury [10].

Early interaction between circulating lymphocytes and the engrafted organ may also initiate the sequence of events mediating the immune response of the host that can eventually result in graft rejection [11]. Thus, we hypothesized that preengraftment depletion of circulating lymphocytes achieved by perioperative RATG administration, in addition to limit the risk of DGF, could also prevent subsequent activation of the immune response and eventual graft rejection [9]. To formally test this hypothesis, we compared, in the setting of a matched-cohort study, the outcomes of 25 consecutive patients who had received a low-dose RATG infusion started before referral to the surgical room with those of 50 gender- and age-matched controls who received the same RATG regimen started after surgery, in combination with the same basiliximab induction and background maintenance immunosuppression. We secondarily explored whether improved outcomes—if any—achieved by peritransplant induction could be explained by early T-cell depletion.

## 2. Materials and Methods

### 2.1. Study Protocol

Since October 2004, all consecutive patients receiving a kidney transplant from deceased donors at the Bergamo Transplant Center were induced with basiliximab in combination with a seven-day course of RATG that was started after surgery, at patient referral to our Nephrology Unit. Since May 2009, the same induction regimen was started before engraftment, as soon as the patient was assigned to receive the transplant. No change was introduced in concomitant maintenance immunosuppressive regimen. Thus, in the present study, we compared clinical outcomes of 21 consecutive patients who received perioperative RATG with those of 42 gender-, age- (±10 years) and treatment- (MMF or AZA) matched reference patients who had received the first RATG dose after surgery. Secondarily, we evaluated changes in circulating T lymphocytes achieved by perioperative RATG infusion to explore the relationship between T-cell counts and clinical outcomes. Outcome data were retrieved from each individual patient up to last available followup and from each couple of corresponding reference-patients throughout the same posttransplant observation period.

Living donor recipients, patients with a peak panel reactive antibody titer >30%, or patients receiving multiorgan transplants were not considered, since they were treated, as per center practice, with a different maintenance immunosuppressive regimen [8]. No changes in selection criteria of donors and recipients, center surgical and medical equips, or surgical and monitoring procedures, were introduced throughout the whole study period.

**Treatment**

Patients received eight-hour daily intravenous infusions of 0.5 mg/kg of RATG (Thymoglobulin, Genzyme corporation, Italy) from the transplant day to day 6 after transplant. The first infusion was started in the Nephrology Unit and was continued during and after the surgical procedure until the administration of the prescribed dose was completed. As previously described [8], the infusion was preceded by the intravenous injection of methylprednisolone (500 mg) and chlorphenamine (10 mg) to prevent acute cytokine release syndrome. The daily doses of RATG were reduced or temporarily withdrew when white blood cell or platelet counts decreased to <2,000/*μ*L or <50,000/*μ*L, respectively. A fixed dose of 20 mg of Basiliximab (Simulect, Novartis, Italy) was administered on day 0 before methylprednisolone infusion and 4 days apart. Methylprednisolone 250 mg were infused on day 1, 125 mg on day 2 after transplant, followed by 75 mg, 50 mg, and 25 mg of oral prednisone administered on posttransplant days 3, 4 and 5, respectively. Thereafter, all subjects were off steroids.

All patients received maintenance immunosuppression with CsA (Sandimmune, Novartis, Italy) given at daily doses targeting blood trough levels between 300 and 400 ng/mL from day 0 to day 7, 200 and 250 ng/mL from day 8 to 30, 150 to 200 ng/mL up to month 6 posttransplant and 100 to 150 ng/mL thereafter. MMF (1.5 g/day; Cell Cept, Roche, Italy) or AZA (125 mg/day if body weight > 75 kg, 75 mg/day if ≤75 kg; Imuran, GlaxoSmithKline, Italy) was administered in a random sequence starting on posttransplant day 1. Doses were reduced if white blood cells and platelet counts decreased to <2,000/*μ*L or <50,000/*μ*L, respectively.

Reference-patients received the same induction and maintenance therapy described above for study patients with the only exception that the first RATG infusion was started postoperatively, when they were referred to the Nephrology Unit (about 30 minutes after the end of the surgery).

**Monitoring and Followup**

All patients and reference-patients were followed according to the same monitoring protocol. Routine clinical and laboratory parameters, including glomerular filtration rate (eGFR) estimated by the Walser equation [12], were evaluated daily up to after transplant day 14, every other day up to month 1, every week up to month 3, and monthly thereafter. Blood CsA concentrations were measured daily up to after transplant day 14 and monthly thereafter.

In patients, circulating T lymphocytes and CD3+CD4+ and CD3+D8+ T cells were counted by fluorescence-assisted cell-sorter analysis before and immediately after the first steroid infusion (i.e., before starting RATG infusion), one hour after the start of RATG infusion, immediately before graft reperfusion, at 10 and 24 hours after the initial sampling, and at 7, 14, 180, and 360 days posttransplant. Reference-patients had the same evaluations before infusion of steroids and RATG, and at 7, 14, 180, and 360 days posttransplant.

**Acute Rejection**

Clinical diagnosis was established on the basis of the following criteria: a transient increase in serum creatinine concentration (>0.3 mg/dL) not obviously explained by cyclosporine trough levels above the target range or by kidney hypoperfusion, vascular thrombosis, or urinary tract obstruction. Clinical diagnosis was confirmed by evidence of renal function recovery following a standard course of intravenous steroid pulses (methylprednisolone 500 mg/day for 3 days, progressively tapered to the maintenance oral dose over 10 days) [8]. A kidney biopsy was performed per protocol to confirm or exclude the clinical diagnosis of acute rejection in those patients with a kidney function that did not recover within 3–5 day of steroid therapy. A bioptic diagnosis of acute rejection was established according to Banff criteria. In all patients who had a kidney biopsy performed to verify a clinical diagnosis of acute graft rejection, the diagnosis was confirmed by the histology findings.

**Delayed Graft Function**

DGF was defined as need for dialysis therapy within the first week after transplant [8]. In DGF patients, initiation of CsA was delayed and steroid treatment was prolonged until serum creatinine spontaneously declined [13]. A graft biopsy sample was taken 7 days after transplant from subjects who remained dialysis dependent to assess the causes of persisting graft dysfunction.

**Cytomegalovirus Reactivation**

Cytomegalovirus (CMV) reactivations were monitored by measuring CMVpp65 antigenemia or CMD DNAemia (since November 2007) by a commercial CMV-PCR method (Cobas Amplicor Monitor) and were treated by intravenous ganciclovir or oral valganciclovir [8].

### 2.2. Statistical Analysis

Baseline recipients' and donors' characteristics were compared between groups by *t*-test, *χ* ^2^, or Fisher's exact test as appropriate. For each parameter, changes over time and differences between groups were assessed by either analysis of variance (ANOVA) for repeated measures or ANOVA factorial, or analysis of covariance (ANCOVA), as appropriate. Time to acute rejection was compared between groups by log-rank test. Univariable analyses were performed to assess whether baseline variables or CsA levels at different times after transplant were significantly associated with the risk of acute rejection. Mechanisms possibly explaining the observed treatment effects on acute rejection were explored by including in the models CsA trough levels during the first week after transplant, considered separately.

Data are expressed as mean ± SD or median (interquartile range) if not otherwise indicated. Statistical analyses were accomplished with SAS software (version 9.1; SAS Institute, Cary, NC). All statistical analyses were by two-tailed tests. The statistical level of significance was *P* < 0.05.

## 3. Results

At transplantation, characteristics of patients and reference-patients and of their corresponding donors were similar, with the only exception of less HLA-B mismatches in patients (Table 1). Twelve (48%) patients and 39 (78%) reference-patients were on MMF therapy (*P* = 0.017). All patients and reference-patients were followedup until the end of the first year after transplant.

### 3.1. Efficacy Outcomes

At study end, all subjects were alive with a functioning graft.

**Allograft Rejection**

**Figure 1:**
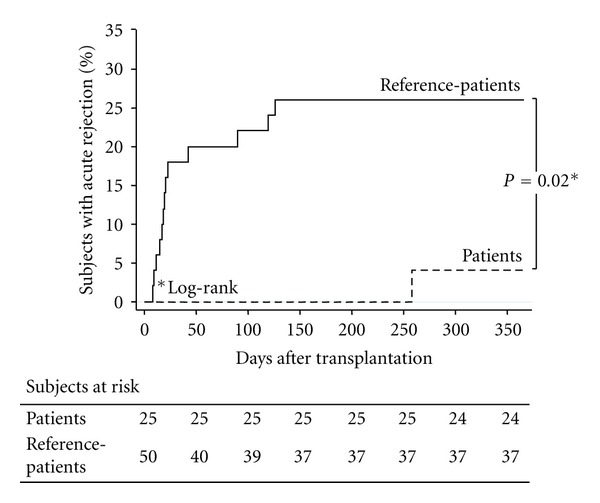
Kaplan-Meier curves of the percentages of patients and reference-patients with acute rejection during the whole follow-up period.

Overall, 14 of the 75 study subjects (18.7%) had one allograft rejection. Rejection episodes occurred in one of the 25 patients and 13 of the 50 reference-patients (4% versus 17.3%, *P* = 0.02, Figure 1). One patient versus six reference-patients received a graft biopsy, since their serum creatinine did not decline after the first steroid pulses. In all these cases, the clinical diagnosis of acute rejection was confirmed by histological analysis and renal function eventually recovered with steroid therapy alone. Banff scores were 1A for the single patient and borderline (*n* = 2), 1A (*n* = 1), 2A (*n* = 1), and 2B (*n* = 2) for the six reference-patients. None of considered donor and recipient characteristics (including the number of mismatches), nor CsA trough levels during the whole follow-up period were significantly associated with the risk of rejection.

**Renal Function Recovery**

Kidney function promptly recovered in all patients, whereas 5 reference-patients required 1 or 2 dialysis sessions because of DGF (*P* = 0.16). eGFR tended to be higher in patients than in reference-patients both at seven days (50.4 ± 24.5 versus 45.6 ± 26.6 mL/min/1.73 m^2^, *P* = 0.44) and at 12 months (56.2 ± 15.3 versus 52.0 ± 14.0 mL/min/1.73 m^2^, *P* = 0.25) after transplant.

**Circulating T Lymphocyte Counts**

**Figure 2:**
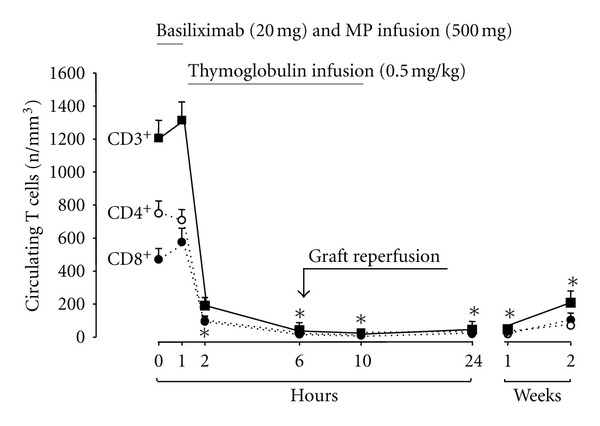
Circulating total T lymphocytes, CD4+ and CD8+ T-cell counts before and at different time points after the start of Thymoglobulim infusion in the 25 patients. All differences between pre- and post-RATG counts were statistically significant (*P < 0.005).

Total circulating T lymphocytes, and CD4+ and CD8+ cells considered separately, did not change appreciably across methylprednisolone infusion. However, they were almost fully depleted over one-hour RATG infusion and no circulating lymphocytes were detectable in any single patient from the sampling preceding the engraftment (approximately after four hours of RATG infusion) up to the one preceding the second RATG infusion on after transplant day 1 (Figure 2). In both groups circulating T cells were still remarkably reduced at two weeks after transplant and showed a similar trend to recover toward normal range over 6–9 months posttransplant (*data not shown*).

### 3.2. Immunosuppressive Therapy

Cyclosporine trough levels were significantly higher in patients than in reference-patients at 3, 4, 5, and 6 days after transplant, but were similar between groups thereafter. The initial difference was fully explained by the fact that in the five reference-patients with DGF, CsA treatment was postponed by an average of 5.8 days. None of these subjects was rejected on subsequent followup. When analyses were restricted to subjects without DGF, CsA trough levels were similar between treatment groups also during the first week after transplant. CsA levels in subjects with or without allograft rejection considered independently from timing of RATG infusion were similar as well. Mean MMF and AZA doses were similar between groups during the whole follow-up period (*data not shown*).

### 3.3. Safety

No cytokine release syndrome symptoms were observed during induction therapy with the only exception of mild and self-limiting fever observed during the first RATG infusion in 17 (34%) reference-patients, but never in patients (*P* = 0.001). Four patients (16%) and 8 reference-patients (16%) had at least one episode of transient leukopenia that always recovered after RATG, MMF, or AZA dose reduction.

The overall incidence of CMV reactivations was significantly lower in patients compared to reference-patients (Table 2), even when analyses were restricted to the 25 patients and 14 reference-patients evaluated by the same diagnostic procedure based on the measurement of CMV DNAemia (Table 2). All reactivations were observed within the first three months after transplant and fully recovered with ganciclovir or valganciclovir therapy.

## 4. Discussion

In our present series of deceased-donor renal transplant recipients, the overall incidence of acute rejections following dual induction therapy with low-dose RATG and basiliximab was remarkably low despite early steroid withdrawal and steroid-free maintenance therapy with low doses of CsA given in combination with MMF or AZA. The novel finding here was that timing of RATG administration in the setting of dual induction therapy remarkably affected posttransplant outcomes. Indeed, only one of the 25 patients receiving perioperative RATG infusion had an acute allograft rejection compared to 13 of the 50 reference-patients receiving RATG infusion after transplant. Moreover, the 5 cases of delayed graft function were all restricted to the reference-patient population. Of note, the incidence of rejections we observed following perioperative RATG administration was amongst the lowest incidences reported in kidney transplantation [3, 14], which is of some relevance considering that this outcome was achieved in the setting of a steroid-free maintenance regimen by RATG cumulative doses that were approximately one-quarter of those employed in most previous series [6, 7] and one-half of those currently recommended in clinical transplantation [3].

We also found that the protective effect against allograft rejection achieved by perioperative RATG administration was associated with a prompt and complete depletion of T lymphocytes from the circulation that ensued over the first hour of RATG infusion and persisted for at least two weeks after transplant. This might have facilitated the establishment of a protolerogenic milieu since, after complete depletion, regenerating alloantigen-specific T cells most likely encountered the graft antigens in a healed state, when they were purportedly reinforced to become anergic [11]. This might explain the effective prevention of allograft rejection we observed in our patients [11]. Indeed, thymoglobulins may facilitate a protolerogenic state through depletional and nondepletional mechanisms [15, 16], involving the modulation of B lymphocyte, dendritic cell, and natural killer T-cell activity [17, 18]. They also promote conversion of CD4+CD25−T cells into CD4+CD25+ regulatory T cells [16]. This effect, however, is at least in part mediated by interleukin 2 (IL-2) activity [19] and might have been limited by concomitant Il-2 inhibition achieved by basiliximab. Actually, *per-protocol* basiliximab coadministration was intended to inhibit those T cells that survived the very low doses of RATG we infused in our subjects. Finding that T lymphocytes were fully depleted from the circulation for at least two weeks after transplantation, *a posteriori* suggests that basiliximab will unlikely complement the antirejection activity of low-dose RATG and might just inhibit RATG-induced expansion of CD4+CD25+ regulatory T cells. This hypothesis merits to be addressed by *ad hoc* trials comparing the antirejection activity of RATG induction with or without concomitant basiliximab therapy.

The protective effect of perioperative RATG infusion against DGF is also worth mentioning. Both RATG and IL-2R antagonists reduce the ischemia/reperfusion injury, possibly by blocking integrin expression on endothelial and T cells and by inhibiting leukocyte adherence to the vessel walls [9, 20–22]. Thus, with dual induction the two drugs could synergistically contribute to accelerate posttransplant renal function recovery. On the other hand, none of the five reference-patients with DGF eventually had acute rejection despite postponed CsA administration. Thus, the excess risk of acute rejection observed with posttransplant compared to perioperative RATG infusion could not be explained by less efficient calcineurin inhibition in reference-patients. This is consistent with evidence that when DGF subjects were not considered in the analyses, CsA through levels were virtually identical between treatment groups throughout the whole observation period.

Of note, early RATG administration did not affect the remarkably good safety profile we previously observed with basiliximab combined to postoperative low-dose RATG infusion [8]. In our present series, no subject had signs of cytokine release syndrome such as chills, hypotension, or severe leukopenia or thrombocytopenia, with the only exception of a mild and self-limiting fever that was observed in some reference-patients during the first RATG infusion, but never in patients. Anesthesia could have masked some cytokine release-related events in the patient cohort. On the other hand, it should be also considered that patients who received pre-transplant RATG administration had the infusion shortly anticipated by steroid administration. Conversely, when RATGs were administered after surgery, the anti-inflammatory effect of pre-transplant steroid infusion was largely vanished, which could explain the increased incidence of fever. Regardless of the above, patients who received pre-transplant RATG infusion had less frequently fever after surgery, which made their post-operative management easier. These findings are of clinical interest, since acute treatment-related adverse events are reported in 40 to 60 percent of patients during infusion of conventional doses of RATG and may require treatment adjustments that may limit the antirejection efficacy of induction therapy [4, 5]. Minimized maintenance immunosuppression, together with the small doses of RATG we administered for induction, might also have contributed to the excellent tolerability of our treatment regimen throughout the whole study period. An unexpected finding of the present study was the significantly lower incidence of CMV reactivations observed in patients compared to reference-patients, even when analysis was restricted to those subjects evaluated by the same diagnostic procedure based on the measurement of CMV DNAemia. Since induction and maintenance immunosuppression regimens were the same for all study subjects, whereas only one patient compared to 13 reference-patients required steroid pulse therapy for the treatment of acute graft rejection, the above findings were most likely explained by reduced patient exposure to steroid, an additional potential benefit of perioperative induction therapy.

The major limitations of our present study were the relatively small sample size and the nonrandomized design. Major strengths were the controlled design with a rigorous matching between patients and reference-patients by pre-defined criteria and the standardized treatment and monitoring guidelines that were applied to all study subjects by the same team at the same institution. These factors altogether contributed to avoid preventable sources of bias. Moreover, serial T-cell counts allowed for the first time to monitor the lymphocytolytic activity of low-dose RATG induction and to explore its clinical implications in the setting of kidney transplantation.

## 5. Conclusions

In renal transplant recipients on a steroid-free maintenance immunosuppressive therapy with low-dose CsA combined to MMF or AZA, the protective effect of dual induction therapy with low-dose RATG and basiliximab against acute allograft rejection and DGF was enhanced when the first infusion of RATG was started before graft reperfusion. Improved outcomes were likely explained by pretransplant depletion of circulating T cells. Finding that prompt and profound T-cell depletion was already achieved during the first pre-transplant RATG administration, combined to evidence that the depleting effect of one single RATG administration is sustained over time [23, 24], provides the background for further studies to assess whether repeated RATG infusions are actually needed to maintain a sustained host hyporesponsiveness against the graft. This is an issue of major clinical relevance since further minimization of induction therapy, combined with minimized maintenance immunosuppression, might help further in improving the risk/benefit profile of antirejection treatment in clinical transplantation. Whether peritransplant administration of very low RATG doses allows preventing acute rejection with minimal immunosuppression independent of add-on basiliximab therapy is the subject of an ongoing further study.

## Figures and Tables

**Table 1:** Baseline characteristics of donors and recipients.

	Overall (*n* = 75)	Patients (*n* = 25)	Reference-patients (*n* = 50)
Donors			
Males (%)	37 (49.3)	12 (48)	25 (50)
Age (yrs)	48.0 ± 13.1	46.9 ± 11.8	48.9 ± 13.8
Weight (Kg)	72.0 ± 14.6	73.3 ± 16.9	71.3 ± 13.5
Mismatches			
A	0.9 ± 0.6	0.8 ± 0.8	0.9 ± 0.5
B	1.3 ± 0.7	1.0 ± 0.6*	1.4 ± 0.7
DR	1.2 ± 0.6	1.0 ± 0.6	1.2 ± 0.6
Recipients			
Males (%)	48 (64)	16 (64)	32 (64)
Age (yrs)	49.3 ± 10.9	51.5 ± 11.4	48.2 ± 10.6
Weight (Kg)	67.4 ± 13.5	70.7 ± 14.3*	65.7 ± 12.9
Cold ischemia time (h)	14.7 ± 7.1	15.7 ± 6.9	14.3 ± 7.2
Causes of ESRD			
Hypertension, renovascular disease	4 (5.3%)	3 (12%)	1 (2%)
Glomerulonephritis	20 (26.7%)	7 (28%)	13 (26%)
Polycystic kidney disease	12 (16%)	4 (16%)	8 (16%)
Other	18 (24%)	7 (28%)	11 (22%)
Unknown	21 (28%)	4 (16%)	17 (34%)

Data are numbers (percent) or mean ± SD. *P < 0.05 versus reference-patients.

**Table 2:** Main adverse events after transplant in the two study groups.

	Patients (*n* = 25)	Reference-patients (*n* = 50)
Acute rejections—*n* (%)	1 (4.0)*	13 (26.0)
DGF—*n* (%)	1 (4.0)	5 (10.0)
N. of dialyses	2	1 (1-2)
Leukopenia—*n* (%)	4 (16)	8 (16)
Thrombocytopenia—*n* (%)	0	1 (2)
CMV reactivations—*n* (%)	5 (20)*	27 (54.0)
*CMV* *DNA *	5/25 (20)	8/14 (57.1)
*Ag* *PP65 *	—	19/36 (52.7)

Data are number (percentage) or median (range). DGF: delayed graft function, leukopenia: leukocytes < 3000/mm^3^, thrombocytopenia: platelets < 50,000/mm^3^, *P < 0.05 versus reference-patients.
